# RETRACTION: Antigenic and Genotypic Similarity between Primary Glioblastomas and Their Derived Neurospheres

**DOI:** 10.1155/jo/9792604

**Published:** 2025-11-21

**Authors:** Journal of Oncology

RETRACTION: V. Caldera, M. Mellai, L. Annovazzi, et al., “Antigenic and Genotypic Similarity between Primary Glioblastomas and Their Derived Neurospheres,” *Journal of Oncology*, 2011, 314962, https://doi.org/10.1155/2011/314962.

The above article, published online on 18 August 2011 in Wiley Online Library (https://wileyonlinelibrary.com), has been retracted by John Wiley & Sons Ltd. The retraction has been agreed due to multiple concerns identified with the figures. Specifically:

• Figures 2e and 2f are identical to panels 3e and 3f in [1].
• The REST bands in the left hand side blots shown in Figure 8a appear identical to the ß-Tubulin bands shown in Figure 4b in [2].
• There are suspected instances of undisclosed splicing in the western blots shown in Figure 8.

The authors did not provide a response to the concerns, and after assessment by the editorial board, it is retracted due to concerns with the reliability of the data.

The authors did not respond to the retraction.
